# Introduction to Editorial Board Member: Professor Francis J. Doyle III

**DOI:** 10.1002/btm2.10054

**Published:** 2017-02-27

**Authors:** Neda Bagheri, Rudiyanto Gunawan

**Affiliations:** ^1^ Chemical and Biological Engineering, Northwestern University, 2145 Sheridan Road, Evanston, IL 60208; ^2^ Department of Chemistry and Applied Biosciences, ETH Zürich, 8093 Zürich Switzerland

In this issue of *Bioengineering and Translational Medicine*, we are delighted to introduce Professor Francis (Frank) J. Doyle III, the Dean of Harvard's Paulson School of Engineering and Applied Sciences. He holds the John A. and Elizabeth S. Armstrong Professor of Engineering and Applied Sciences. Prof. Doyle received his BS degree in Chemical Engineering from Princeton University, his MS degree from Cambridge University (UK), and his PhD degree from the California Institute of Technology.

Prof. Doyle started his academic career in 1992 at Purdue University in the Department of Chemical Engineering. In 1997, Prof. Doyle moved to the Department of Chemical Engineering at the University of Delaware, initially as an associate professor, before becoming a full professor. Between September 2001 and June 2002, he was a Humboldt Research Fellow at the Institute for Systems Theory and Automatic Control at the University of Stuttgart, Germany. In 2002, Prof. Doyle moved to the University of California Santa Barbara (UCSB). At UCSB, Prof. Doyle held the Duncan and Suzanne Mellichamp Endowed Chair in Process Control. He also began a very fruitful partnership with the Sansum Diabetes Research Institute in Santa Barbara as an Adjunct Senior Investigator. His group was the first to demonstrate the operation of a fully closed and completely automated glucose feedback loop in a clinical trial in diabetic patients.

In addition to research and teaching excellence, Prof. Doyle has had a passion for administrative leadership. At UCSB, he held several administration positions. He (co)directed the Institute for Collaborative Biotechnologies, a UCSB/MIT/Caltech Army sponsored University Affiliated Research Center. Under his leadership, ICB emerged as a globally leading research organization for biologically‐inspired technological innovation. Prof. Doyle also served as the chair of the Chemical Engineering department and Associate Dean of Research in the College of Engineering at UCSB. In the latter role, he championed building of UCSB's bioengineering research enterprise and infrastructure. In July 2015, Prof. Doyle moved to Harvard University as Dean of Paulson School of Engineering. Currently, Dean Doyle is realizing his vision of the new phase of Harvard's School of Engineering and Applied Sciences on the back of interdisciplinary bridges with Harvard's Business and Medical Schools.

At the beginning of his career, Prof. Doyle's research focused primarily on the applications of advanced control schemes on nonlinear, multivariable, constrained industrial processes, with emphasis on particulate systems, and pulp and paper. At Purdue, he met and began a fruitful collaborative research project on glucose control for Type 1 Diabetes with Prof. Nicholas Peppas (an Editorial Board Member). While their paths later diverged, the collaboration seeded a transition in Prof. Doyle's research portfolio toward biology and biomedical applications. In the past 15 years, Prof. Doyle and his group have created and applied sophisticated tools from control systems theory to analyze the regulatory mechanisms of biological systems with the end goal of being able to manipulate their behaviors. The research has led to impactful advances in the understanding and control of biological systems, particularly in the regulation of blood glucose and circadian rhythms.

Over the past two decades, the Doyle group has been at the forefront of the design and synthesis of control algorithms for the development of the artificial pancreas (AP) for type 1 diabetes treatment. In 1999, Prof. Doyle published a seminal paper on model predictive control (MPC) of blood glucose (BG) with his former PhD student, Prof. Robert Parker of U Pittsburgh, and Prof. Nicholas Peppas.^1^ Unlike the classical proportional‐integral‐derivative (PID) controller that acts in response to departures from the desired process set point, MPC can anticipate changes in the process outputs (e.g., BG level) and prescribe the optimal control action (e.g., delivery of insulin). Following Prof. Doyle's pioneering work, many MPC algorithms for the AP have been developed, including landmark contributions from the Doyle group.^2^ As evidence of Prof. Doyle's impact in medicine, MPC has become one of the mainstream control algorithms used in closed‐loop BG control in clinical studies. Notably, Prof. Doyle's control algorithms have been successfully validated in clinical settings^3^ and—with support from the National Institutes of Health—are part of the largest long‐term clinical trial for the AP system.^4^ His algorithms are being used in over 10 sites worldwide, and over 700 human subjects have undergone closed‐loop testing in the clinics around the world based on these algorithms.

**Figure 1:**
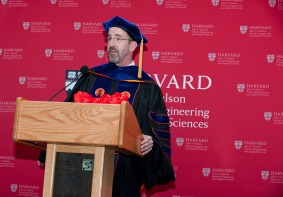
Professor Doyle addresses students at the Harvard John A. Paulson School of Engineering and Applied Sciences graduation ceremony in June 2016

In addition to diabetes, Prof. Doyle's contribution to circadian biology research has generated major insights on the design principles of the biological network behind the extremely robust body clock. In humans, the central pacemaker of the circadian rhythm comprises ∼20,000 neurons in the suprachiasmatic nucleus (SCN) region of the hypothalamus, working in synchrony to generate a coherent circadian signal. Within each neuron, a gene regulatory network with interlocking feedback loops generates the endogenous oscillations of circadian protein expression. Using systems modeling and analysis of this network, Prof. Doyle and his coworkers were able to show how robustness in circadian periodicity and phase response is accomplished by allocating the fragility of the regulatory network to tightly regulated modules (e.g., the maximum gene transcription rate).^5^ Based on the understanding of this robust‐yet‐fragile regulatory network, the Doyle group was further able to optimally manipulate (or entrain) the circadian rhythm to a desired reference cyclic behavior using MPC algorithms.^6^ Recent work by Prof. Doyle and his collaborators shed new light on the functional network and significance of intercellular coupling among SCN neurons, including the spatial organization of these neurons in the SCN,^7^ their synchronization,^8^ and mutation buffering.^9^ Novel insights from Prof. Doyle's research elucidate key challenges in circadian biology and, more generally, advance our understanding of biological oscillators, such as annual coral spawning,^10^ so we can accurately predict and potentially modulate their complex dynamics.

Prof. Doyle's research has been disseminated in over 300 journal articles, book chapters and books, garnering over 18,500 citations. In recognition of his research achievements, Prof. Doyle has received numerous awards, including his election to the National Academy of Medicine (NAM) in 2016. He also received AAAC Control Engineering Practice award in 2015 and the Computing in Chemical Engineering award from AIChE in 2005. He is an elected Fellow of the American Association for the Advancement of Science (AAAS), the International Federation of Automatic Control (IFAC), the American Institute for Medical and Biological Engineering (AIMBE), and the Institute of Electrical and Electronics Engineers (IEEE). Prof. Doyle has also been an influential leader in many renowned scientific communities. He currently serves as vice president of IFAC, and previously served as President of IEEE Control Systems Society, President of Computer Aids in Chemical Engineering (CACHE), and Director and Chair of the Computing & Systems Technology Division within the American Institute of Chemical Engineers (AIChE).

**Figure 2:**
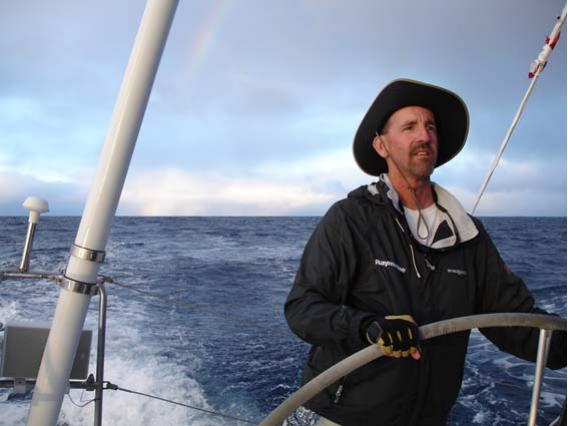
Professor Doyle navigates the Pacific Ocean over several days as he and his crew compete in the Transpacific Race from California to Hawaii in 2011

In addition to his commitment to research, Prof. Doyle is a passionate educator and has contributed greatly to the process control pedagogy of chemical engineers. He has published several books in the control field, and is a coauthor of the widely‐adopted Process Dynamics and Control textbook (with Prof. Seborg and Mellichamp, UCSB, and Prof. Edgar, UT Austin). His accomplishments as an educator have been recognized through the Ray Fahien award from the American Society for Engineering Education (ASEE) and the ASEE Section Outstanding Teacher Award (Illinois/Indiana).

Beyond science, engineering, and medicine, Prof. Doyle invests time in three additional pillars: his family, soccer, and sailing. Prof. Doyle is a proud father and husband and enjoys spending time with his family exploring the outdoors. He is also certified as a professional soccer referee and dedicates many weekends on the field keeping up with college level athletes. Throughout his life, Prof. Doyle has embraced sailing both recreationally and competitively. One of his most memorable competitions included a regatta from Los Angeles to Hawaii (the TransPac), where he put to practice his expertise in circadian biology to maximize the performance and experience of his team around the clock and across time zones.

As former members of the Doyle group, we benefitted tremendously from the collegial, collaborative and interdisciplinary research atmosphere that Prof. Doyle cultivated in his laboratory. His positive energy is quite infectious and brought out the best in us. Despite his busy schedule and countless commitments, Prof. Doyle was always eager to discuss research and champion each and every one of our projects. He has always had an open and positive outlook, embraced change, and served as an advocate for future generations of scientists. We feel most fortunate to have had the invaluable opportunity to work with, and learn from, Prof. Doyle as a mentor, teacher, role model, and colleague. His balance and leadership have always been an inspiration. We look forward to embracing and extending his teaching in our own academic pursuit.


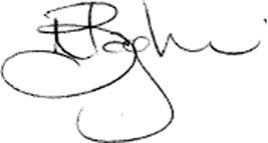
 
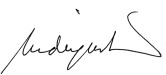


Neda Bagheri^1^, Rudiyanto Gunawan^2^

^1^ *Chemical and Biological Engineering, Northwestern University, 2145*

*Sheridan Road, Evanston, IL 60208*

^2^ *Department of Chemistry and Applied Biosciences, ETH Züurich, 8093*

*Züurich Switzerland*
